# Rapidly progressing necrotic hand ulcer in a patient with angioimmunoblastic T‐cell lymphoma

**DOI:** 10.1002/ski2.236

**Published:** 2023-04-15

**Authors:** Hua Xian Elizabeth Wang, Xue Ting Ooi, Kok Hing Lim, Choon Chiat Oh

**Affiliations:** ^1^ Department of Dermatology Singapore General Hospital Singapore Singapore; ^2^ Division of Dermatology Department of Medicine National University Hospital Singapore Singapore; ^3^ Department of Pathology Singapore General Hospital Singapore Singapore

## Abstract

Mucormycosis is a fungal infection caused by opportunistic fungi of the phylum Glomeromycota, subphylum Mucormycotina. In developed countries, it affects patients with haematological malignancies undergoing chemotherapy and those who have received allogenic stem cell transplants, while in developing countries it is seen in those with uncontrolled diabetes mellitus. Herein, we report a case of cutaneous mucormycosis in a 67yo Chinese gentleman with background of angioimmunoblastic T cell lymphoma (AITL) on chemotherapy. We also share the clinicopathological findings of this and correlate these findings with those present in the current literature. Finally, we outline treatment options and prognosis of cutaneous mucormycosis.

## Graphical abstract

A 69‐year‐old Chinese male with background angioimmunoblastic T cell lymphoma (AITL) on chemotherapy, presents with a sudden onset large necrotic ulcer over the dorsum of his right hand. He was diagnosed with cutaneous mucormycosis and the cause is suspected to be due to direct inoculation from intravenous plug insertion over his right hand dorsum.

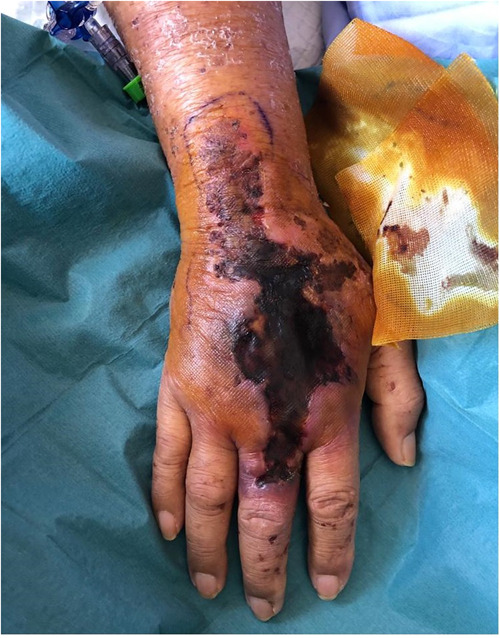

## 1 CLINICAL FINDINGS

**FIGURE 1:**
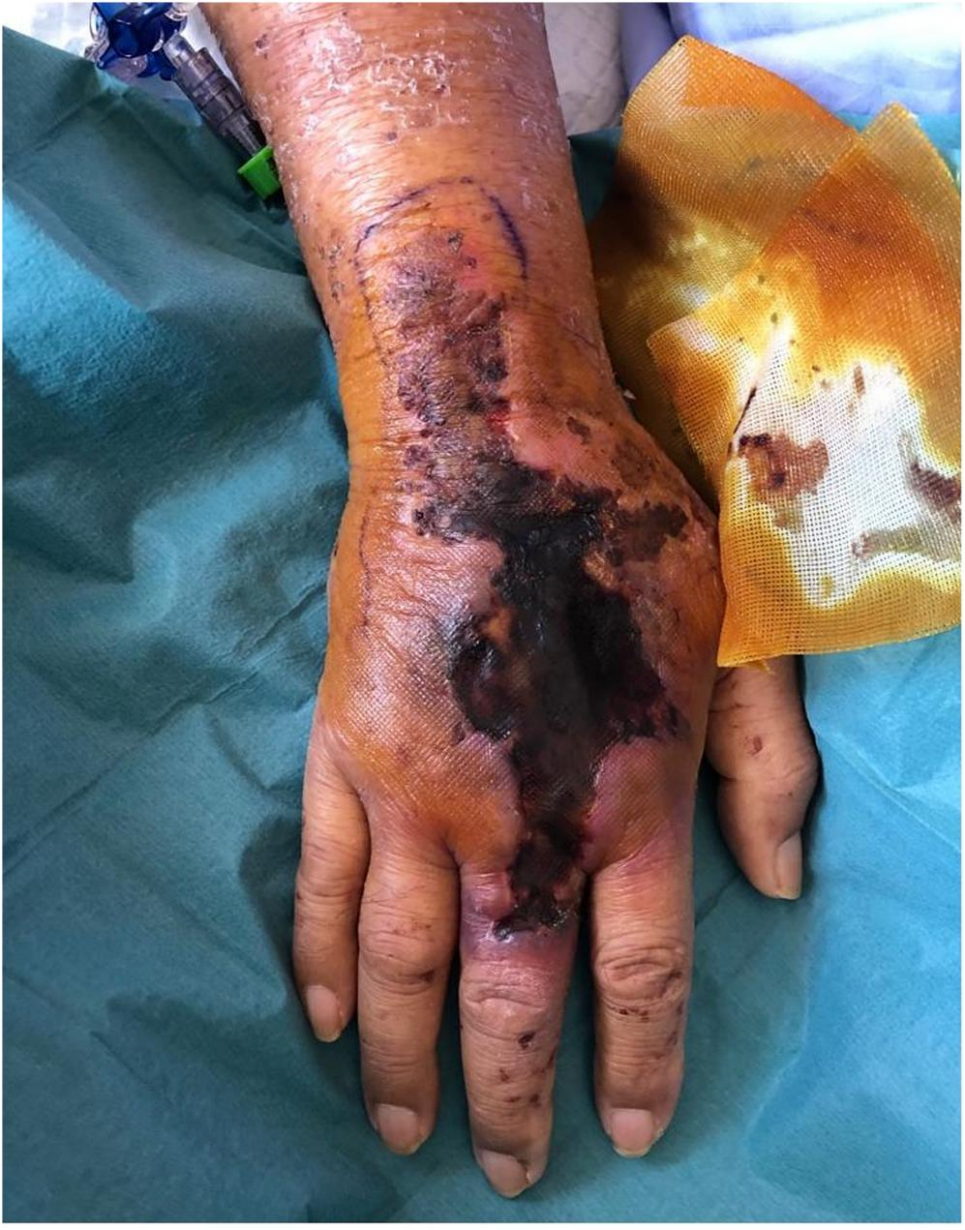
Necrotic ulcer with surrounding retiform purpura over right dorsum of hand.

A 69‐year‐old Chinese male presents with a sudden onset large necrotic ulcer over the dorsum of his right hand. He has a background of newly diagnosed angioimmunoblastic T cell lymphoma (AITL) on chemotherapy. The ulcer began as an erythematous patch that progressed rapidly into a painless ulcer. On examination, a 10 cm necrotic ulcer with surrounding retiform purpura is seen over the right dorsum of hand (Figure 1).

## 2 HISTOPATHOLOGICAL FINDINGS

**FIGURE 2:**
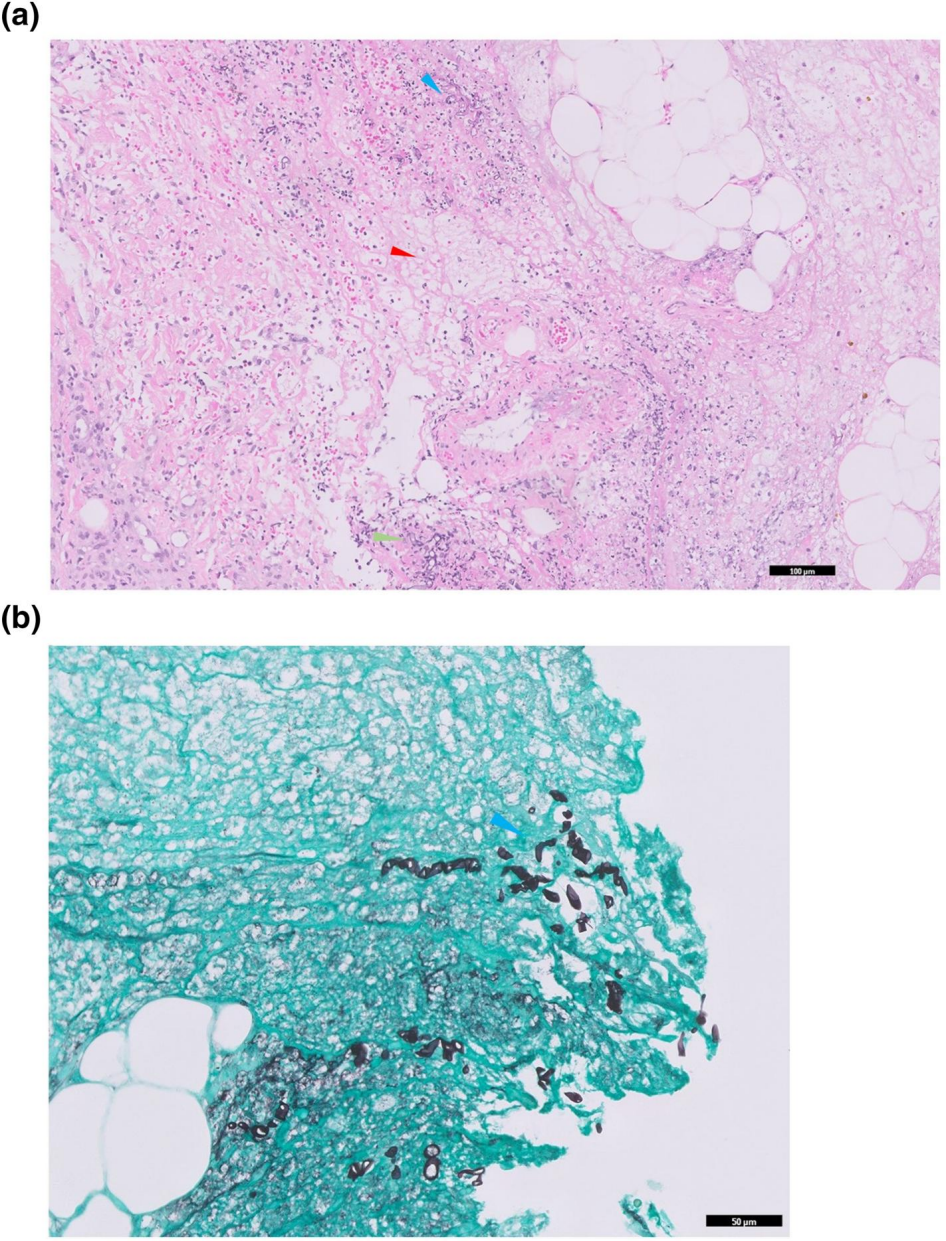
(a) Prominent necrotic changes (red arrowhead) in the deep dermis and subcytis with accompanying predominantly neutrophilic infiltrate (green arrowhead), amidst hyphae of variable thickness and right‐angle branching (blue arrowhead). Haematoxylin‐eosin stain; original magnification: x10, (b) Hyphae of variable thickness and right‐angle branching (blue arrowhead). Grocott's Methenamine Silver stain; original magnification: x20.

Histology revealed prominent necrotic changes particularly towards the deep dermis and subcutis with a predominantly neutrophilic infiltrate (Figure 2a.) amidst hyphae of variable thickness and right‐angle branching (Figure 2b.). These were positive for Periodic acid‐Schiff stain (PAS) and Grocott's methenamine silver stain (GMS). The epidermis was acanthotic with overlying keratotic crust containing entrapped fungi. There were no granulomas, vasculitis or atypical cells seen.

## 3 DIAGNOSIS

Cutaneous mucormycosis.

## 4 DISCUSSION

Mucormycosis is a fungal infection caused by opportunistic fungi of the phylum Glomeromycota, subphylum Mucormycotina. In developed countries, it affects patients with haematological malignancies undergoing chemotherapy and those who have received allogenic stem cell transplants, while in developing countries it is seen in those with uncontrolled diabetes mellitus. Other common risk factors include solid organ transplantation, deferoxamine therapy, drug injections, renal failure, infant low birth weight, malnutrition, HIV infection, systemic lupus erythematosus, burns, trauma, aplastic anaemia, and steroid use.^1^ Based on organs affected, mucormycosis can be classified as one of 6 forms: rhinocerebral, pulmonary, cutaneous, gastrointestinal, disseminated, and uncommon presentations.^2^

Cutaneous mucormycosis usually occurs because of direct inoculation of the fungal spores onto the skin. Dissemination from other organs can occur as well but is rare.^2^ Clinical presentation typically begins with an erythematous to purple indurated plaque that eventually becomes necrotic and progress into an eschar. This can be gradual in onset or fulminant. Other presentations can include purpuric lesions, tender nodules, swollen and scaly plaques.

Our patient is a classic example of cutaneous mucormycosis in an immunocompromised patient. His main risk factor is his background AITL on chemotherapy which made him susceptible to this opportunistic infection. The cause is suspected to be due to direct inoculation from intravenous plug insertion over his right hand dorsum.

Diagnosis is made by performing a biopsy of the lesion, ideally down to the subcutaneous tissues. Examination under the microscope should yield distinct findings of Mucorales such as thick, hyaline, non‐septated and bi‐furcated hyphae which may be seen with haematoxylin and eosin stain but are best visualised with PAS and GMS staining. Other common histological findings are necrosis, an inflammatory infiltrate consisting of polymorphonuclear cells, plasma cells and eosinophils, thrombosis, oedema and infarctions.^1^ A sample should also be sent for fungal cultures.

Treatment of cutaneous mucormycosis includes surgical debridement and systemic antifungals. Reducing risk factors such as withholding immunosuppressants and optimising diabetes control is also helpful in managing the disease. Other complementary treatment include hyperbaric oxygen to prevent systemic spread and improve surgical and medical treatment.^3^ In terms of prognosis, cutaneous mucormycosis has better prognosis than any other systemic forms of mucormycosis, with mortality rates of around ~16%, compared to 100% in disseminated mucormycosis.^4^ Therefore, early treatment is imperative to prevent spread and worse prognosis.

In the case of our patient, a skin biopsy was done, and tissue samples were sent for fungal and aerobic cultures. A CT thorax was done which showed findings of a new left lower lobe consolidation with worsening pleural effusions. Due to concerns for disseminated mucormycosis, our patient was started on treatment with IV Amphotericin B. The eventual tissue fungal culture grew *Rhizopus arrhizus*, sensitive to Amphotericin B. He subsequently succumbed to his underlying malignancy and passed away.

## AUTHOR CONTRIBUTIONS

**Hua Xian Elizabeth Wang**: Conceptualization (Equal); Data curation (Equal); Formal analysis (Equal); Writing – original draft (Equal); Writing – review & editing (Equal). **Xue Ting Ooi**: Conceptualization (Equal); Data curation (Equal); Formal analysis (Equal). **Kok Hing Lim**: Investigation (Equal); Methodology (Equal); Validation (Equal); Visualization (Equal). **Choon Chiat Oh**: Conceptualization (Equal); Data curation (Equal); Formal analysis (Equal); Investigation (Equal); Methodology (Equal); Supervision (Equal); Validation (Equal); Writing – original draft (Equal); Writing – review & editing (Equal).

## CONFLICT OF INTEREST STATEMENT

None to declare.

## ETHICS STATEMENT

Not applicable.

## Data Availability

The data that support the findings of this study are available from the corresponding author upon reasonable request.
